# Innovative Artificial-Intelligence- Based Approach for the Biodegradation of Feather Keratin by *Bacillus paramycoides*, and Cytotoxicity of the Resulting Amino Acids

**DOI:** 10.3389/fmicb.2021.731262

**Published:** 2021-10-22

**Authors:** Zeiad Moussa, Doaa B. Darwish, Salma S. Alrdahe, WesamEldin I. A. Saber

**Affiliations:** ^1^Microbial Activity Unit, Microbiology Department, Soils, Water and Environment Research Institute, Agricultural Research Center, Giza, Egypt; ^2^Botany Department, Faculty of Science, Mansoura University, Mansoura, Egypt; ^3^Department of Biology, Faculty of Science, University of Tabuk, Tabuk, Saudi Arabia

**Keywords:** keratinase, bioremediation, biodegradation, artificial neural network, mineral salts, artificial intelligence, response surface methodology

## Abstract

The current study reported a new keratinolytic bacterium, which was characterized as *Bacillus paramycoides* and identified by 16S rRNA, and the sequence was then deposited in the GenBank (MW876249). The bacterium was able to degrade the insoluble chicken feather keratin (CFK) into amino acids (AA) through the keratinase system. The statistical optimization of the biodegradation process into AA was performed based on the Plackett–Burman design and rotatable central composite design (RCCD) on a simple solid-state fermentation medium. The optimum conditions were temperature, 37°C, 0.547 mg KH_2_PO_4_, 1.438 mg NH_4_Cl, and 11.61 days of incubation. Innovatively, the degradation of the CFK process was modeled using the artificial neural network (ANN), which was better than RCCD in modeling the biodegradation process. Differentiation of the AA by high-performance liquid chromatography (HPLC) revealed the presence of 14 AA including essential and non-essential ones; proline and aspartic acids were the most dominant. The toxicity test of AA on the HepG2 cell line did not show any negative effect either on the cell line or on the morphological alteration. *B. paramycoides* ZW-5 is a new eco-friendly tool for CFK degradation that could be optimized by ANN. However, additional nutritional trials are encouraged on animal models.

## Introduction

The poultry industry is one of the sectors that have been subjected to various improvements but still has a critical problem, represented in poultry feather waste. Therefore, an important step may need to be taken to manage such a problem. The poultry industry is a worldwide important sector, through which, suitable-priced meat and eggs are produced. According to Food and Agriculture Organization (FAOSTAT, 2018), chickens alone represent 90% of the poultry industry in the world. Such an industry generates a considerable mass of wastes. Feathers are an important constituent of such wastes, representing 5–10% of the weight of the chicken. Therefore, billions of tons of feathers are generated all over the world that causes a real environmental challenge (Li, 2021). That is why the ideal management of such vast amounts of feathers is of great concern.

As an insoluble protein, keratin is the main component of chicken feathers (91%), while lipids represent 1% and water represent 8% (Saravanan and Dhurai, 2012). Keratins are stable, fibrous, insoluble, and structural proteins (Adelere and Lateef, 2019). Besides being non-eco-friendly, the physical and chemical methods of feathers treatment are money-energy consuming, and can, further, destroy some essential amino acids (AA). Alternatively, keratinolytic microorganisms are an effective tool for the degradation of feather keratin into a simpler form. In this respect, keratinolytic bacteria is a good candidate for feathers’ degradation. This biological-based technique has many advantages; it is an eco-friendly, cheap, and simple method. Importantly, several bioactive compounds are produced, besides AA, such as keratinase enzyme, proteins, and peptides (Adelere and Lateef, 2019; Tamreihao et al., 2019).

The biological degradation of feather keratin leads to the production of several valuable AA, mainly composed of serine, proline, cysteine, and glutamine (Saravanan and Dhurai, 2012). Bioconversion of keratin into AA improves the nutritional value and protein content of animal diets. Functions of AA beyond protein synthesis must be considered in dietary formulations to boost and support the immune system, improve the efficiency of nutrients, growth, development, reproduction, lactation, and wellbeing in animals as well as advance the general health condition of the organism (Hasabi et al., 2014; Kelly and Pearce, 2020).

Economically, the biodegradation of keratin is expected to reduce the production cost, and yield a high amount of AA. Notably, the safe disposal of such biomass residual will positively reflect on the environment. The end products of bacteriological degradation can be used in many fields, such as animal feedstuff, plant fertilizers, medical and pharmaceutical applications, cosmetics and leather, detergents, biocatalysts industries, and others (Tesfaye et al., 2017). Furthermore, using a simple fermentation medium is found to economize the biodegradation process; in this respect, the inorganic salts in small amounts may promote the bacteria population to multiply, and this is apparently due to the different toxic mechanisms of these compounds at high concentrations (Korkeala et al., 1992; Miettinen et al., 1997). However, inorganic salts were found to exert a positive or negative effect on the biodegradation of keratin (Ni et al., 2011). So, it is worthy to explore the effect of such salts on keratinolytic behavior.

Keratin is an insoluble protein resistant to degradation by common proteolytic enzymes. Microbial keratinases (EC 3.4.21/24/99.11) are special proteolytic enzymes that can break down the insoluble keratin such as feather, into peptides and AA in a simple way. The enzymes are also characterized by their high substrate specificity (Saber et al., 2010; Tamreihao et al., 2019).

Therefore, exploring efficient new keratinolytic bacteria is an essential step in the bioprocessing of the keratin waste. Besides, the use of a biological-based degradation strategy of feather waste into AA, keratin will be turned from being an environmental problem into eco-friendly and valuable products, i.e., AA, which have additional beneficial applications in many industries. Due to the growing interest of such AA, an important role in growth, health, and disease prevention in humans, animals, and plants was revealed, by which AA, e.g., proline, modulates gene expression, enhances the growth of the small intestine and skeletal muscle, or reduces excess body fat, as well as healing the metabolic diseases, e.g., obesity, diabetes, and cardiovascular disorders (Siddique et al., 2018; Di Fiore et al., 2019; Zhang et al., 2020).

Both solid-state fermentation (SSF) and response surface methodology (RSM) are selected as the fermentation technique during the bioconversion process. SSF has ecological and economic advantages, e.g., yields a higher amount of the target product, low energy requirements, and lower wastewater production (Saber et al., 2021). So, it has been selected in the current study, since very few studies used such fermentation techniques in the biodegradation of feather keratin.

Regarding the experimental design, the RSM and artificial neural network (ANN) were used to optimize and model the degradation process. RSM introduces several advantages over the ordinary design of the one-factor (variable)-at-a-time. RSM is a useful statistical mathematical method used for constructing, analyzing, and optimizing the experimental conditions by finding the analytical relationship between the input and output variables considered in the fermentation medium. RSM can also produce precious mathematical modeling functions to determine optimal conditions using a smaller amount of data and fewer numbers of experimental runs (Rodrigues et al., 2013; Saber et al., 2015).

The ANN is an alternative categorical modeling approach with higher accuracy, in generating mathematical relationships between input and output variables. ANN can superbly replace the other modeling approaches, such as RSM. Besides having advantages of the elucidation of the behavior of the bioprocess parameters, the ANN model can, hypothetically, deal with the complex non-linear relationships more truthfully because it includes all data points of an experiment while building the model (Saber et al., 2021). Other benefits of ANN include being more accurate than other modeling methods, which means that ANN is quite robust to noise in the training data, even if the training examples contain errors, which do not affect the final output. Furthermore, unlike other modeling techniques that fail to accurately describe object functions as the number of variables increases and interactions become too complex, ANN successfully addresses and overcomes such limitations. ANN can handle noisy data, mimic biological neural networks, outperform statistical/regression-based models, and accurately predict and model highly non-linear and complex biological processes (Haque et al., 2016; Elsayed et al., 2021; Saber et al., 2021).

The modeling system of ANN includes the choice of the architecture, picking of the hidden layer(s) and their neurons, then learning, and training of the created ANN, and, finally, validation of the developed model (Ram Talib et al., 2019). To the best of our knowledge, this is the first report on using ANN in the biodegradation of keratin wastes.

The current investigation was initiated for discovering a novel keratinolytic microorganism and innovative modeling (using artificial intelligence) of a simple medium for biodegradation of feather keratin into AA. Confirmatory, the AA biosynthesized were evaluated for their cytotoxicity.

## Materials and Methods

### Bacteria and Inoculum Preparation

The bacteria used were selected from a collection of bacteria that had previously been isolated and purified from spoiled feathers. The six bacterial isolates were chosen based on their ability to grow and produce clear zone on keratin-agar medium (Parashar et al., 2017).

For inocula preparation, a bacterial cell suspension was prepared by adding 10 ml of sterilized distilled water to a 24-h-old bacterial culture grown on slants of nutrient glucose agar medium. Then, the grown cells were scrapped and the final cell count was adjusted by a hemocytometer to 10^8^ cfu/ml.

### Chicken Feather Powder

White chicken feathers of white chicken were washed and cut into small fragments then dried in a ventilated oven at 40°C for 72 h. The feather powder was prepared by milling and the coarse particles were removed; the feather powder was used as the fermentation substrate.

### Culturing Technique and Fermentation Conditions

SSF was applied during the bacterial fermentation process on CFK. Bacteria were grown on CFK as a fermented substrate. The medium of Cai et al. (2008) was used with some modification. A weight of 5 g of CFK was placed in 500-ml Erlenmeyer flasks, supplemented with 5 ml of a stock solution composed from (g/L) K_2_HPO_4_ (1.4), KH_2_PO_4_ (0.7), NaCl (0.5), NH_4_Cl (1.0), MgSO_4_.7H_2_O (0.1), and CaCl_2_.2H_2_O (0.01), with the pH adjusted to 7.5. The medium was autoclaved at 121°C for 15 min. Inoculation was carried out using 5 ml from the abovementioned inoculum.

Unless otherwise specified, the culture was grown at 35°C for 14 days. During the fermentation process, the moisture was kept at 65% by adding distilled water when needed. After incubation, 50 ml of distilled water containing 0.01% Tween 80 was added to each flask, shaken for 30 min at 150 rpm, and filtered through Whatman No. 1 filter paper, and then centrifuged at 5,000 rpm for 15 min. The biodegradation process was evaluated by measuring the keratinolytic activity (KA) (keratinase) and AA released in the collected supernatant.

The KA of the six bacterial isolates was screened on the SSF medium under the fermentation conditions reported above. Accordingly, the potent keratinolytic strain was selected for further studies.

### Biochemical Tests

#### Keratinase Assay

KA was measured with soluble keratin as a substrate. Soluble keratin (0.5%, w/v) was prepared from white chicken feathers (Wawrzkiewicz et al., 1987). Keratinase activity was then determined by incubating the reaction mixture (1 ml crude enzyme and 1 ml soluble keratin) in a water bath at 50°C for 10 min, and the reaction was stopped by adding 2.0 ml of trichloroacetic acid (TCA) (Cai et al., 2008). The control contains the control filtrate and keratin solution. One unit (U) of KA was defined as the increase by 0.01 of the absorbance at A_280_ nm using a UV-VIS spectrophotometer (Jenway 7315) for 0.01 per minute per gram of fermented CFK under the conditions described above.

#### Determination of Amino Acids in the Fermented Medium

The same previous method (Cai et al., 2008) was used to measure the amount of AA in the fermented medium. Equal amounts of culture supernatant and 10% TCA were mixed, followed by centrifugation at 5,000 rpm for 15 min to obtain a clear filtrate, and the absorbance was measured at A_280_ nm using a UV-VIS spectrophotometer (Jenway 7315). Using tyrosine as a standard, the amount of released AA monomers was calculated in micrograms per gram of fermented CFP. Uninoculated medium subjected to the same fermentation conditions was served as a control.

### Bacterial Identification

#### Morphological and Microscopic Identification

The selected bacterial isolate was subjected to the standard morphological and biochemical tests, followed by scanning electron microscopy (SEM) investigation. The surface of bacterial cells was gold-coated and examined at various magnifications using SEM and JEOL TEM-2100 attached to a CCD camera at an accelerating voltage of 200 kV.

#### 16S rRNA Sequencing

The most active keratinolytic bacterial isolate detected in the above screening was identified on a molecular basis. Extraction of bacterial DNA was conducted based on the method of Sharma and Kango (2021), with some modifications, by mixing 200 μl of bacterial cell suspension thoroughly with 95 μl of water, 95 μl of solid tissue buffer, and 10 μl of proteinase K, in a microcentrifuge tube, and then incubated at 55°C for 2 h before centrifugation at 12,000 × *g* for 1 min. The aqueous supernatant was transferred to a clean tube (300 μl), to which 600 μl of Genomic Binding Buffer was added. The mixture was transferred to a Zymo-Spin^TM^ IIC-XL Column. The collection tube was centrifuged (≥12,000 × *g*) for 1 min, and the flow-through was discarded. DNA re-Wash Buffer (400 μl) was added to the column in a new collection tube and centrifuged again at 12,000 × *g* for 1 min. Then, 700 μl of g-DNA Wash Buffer was added and centrifugated, at 12,000 × *g*, for 1 min. Another 200 μl of g-DNA Wash Buffer was added, followed by centrifugation (12,000 × *g*) for 1 min, and the collection tube was discarded. Finally, 3 μl of elution buffer was added and the mixture was incubated for 5 min and then centrifugated (12,000 × *g*) for 1 min.

The polymerase chain reaction (PCR) was performed in a reaction mixture, containing 25 μl of MyTaq Red Mix, 8 μl of DNA Template (change), 1 μl (20 pmol) each of forward 16S rRNA primer 27f (5′-AGAGTTTGATCMTGCCTCAG-3′) and reverse 16S rRNA primer 1492r (5′-TACGGYTACCTTGTTACGACTT-3′), and 15 μl of Nuclease Free Water. The PCR was accomplished using a gradient thermocycler, during which 6 min initial denaturation at 94°C, followed by 35 amplification cycles of 1 min at 94°C, 1 min of annealing at 56°C, and 1 min of extension at 72°C, followed by a 5-min final extension at 72°C. The amplified DNA fragments obtained from PCR were then analyzed *via* agarose gel electrophoresis 1% (w/v), and the remaining mixture was purified using QIA quick PCR purification reagents (Qiagen, United States). The purified PCR product was sequenced, by using two primers, and resolved on an Applied Biosystems model 3730XL automated DNA sequencing system (Applied BioSystems, United States) and deposited in the GenBank database. The sequence analyzed using BLASTn was aligned with the corresponding 16S rRNA sequences of the type strains retrieved from the GenBank databases.^1^ Twenty aligned sequences with more than 99% similarity were selected for multiple alignments and phylogenetic analysis using the neighbor-joining method, fitting to the distances of Jukes-Canto. The confidence level of each branch was calculated by 1000 bootstrap replicates. All ambiguous positions were removed for each sequence pair (pairwise deletion option). There was a total of 497 positions in the final dataset. Evolutionary analyses were conducted using the MEGA 10 software package (Kumar et al., 2018).

### Optimization and Modeling

For optimization of the biodegradation process, the medium salts, as nutritional parameters, were investigated. The optimization and modeling procedure were carried out through statistical design approaches, including (1) screening and selecting the significant salts by the experimental design of Plackett–Burman (PBD), (2) optimization of the selected salts by the rotatable central composite design (RCCD), and (3) determination of the most fitted model for the biosynthesis of AA using RCCD and ANN.

#### Screening of the Tested Medium Salts by Plackett–Burman

The screening trial aids in the identification of relevant parameters that contribute significantly to the bioprocess CKF into AA, as well as reduce the process costs, by filtering out insignificant (noise) parameters and avoiding costly research on comparatively unimportant ones. The fermentation process was performed on CFK supported by different levels of the tested salts of the previous medium at higher (+1) and lower (−1) concentrations (Table 1). According to the design procedure, the relationship between the actual value and the coded values of the tested parameters is calculated by the following equation:

**TABLE 1:** The Plackett–Burman design with the actual (mg/g CFK) and codified values of the tested medium components of *B. paramycoides* ZW-5 and the corresponding keratinolytic activity and amino acids.

**Trial**	K_2_HPO_4_		KH_2_PO_4_		NaCl		NH_4_Cl		MgSO_4_		CaCl_2_		AA (μg/g CFK) ± SD	
	Actual	Coded	Actual	Coded	Actual	Coded	Actual	Coded	Actual	Coded	Actual	Coded	Response	Predicted
1	1.8	1	0.4	−1	0.7	1	0.5	−1	0.05	−1	0.005	−1	490.014.0	370.3
2	1.8	1	1.0	1	0.3	−1	1.5	1	0.05	−1	0.005	−1	760.08.5	762.7
3	1.0	−1	1.0	1	0.7	1	0.5	−1	0.15	1	0.005	−1	104.313.8	125.7
4	1.8	1	0.4	−1	0.7	1	1.5	1	0.05	−1	0.015	1	1,209.116.5	1305.5
5	1.8	1	1.0	1	0.3	−1	1.5	1	0.15	1	0.005	−1	712.112.6	709.3
6	1.8	1	1.0	1	0.7	1	0.5	−1	0.15	1	0.015	1	93.36.5	159.4
7	1.0	−1	1.0	1	0.7	1	1.5	1	0.05	−1	0.015	1	1,168.717.5	1114.2
8	1.0	−1	0.4	−1	0.7	1	1.5	1	0.15	1	0.005	−1	1,228.021.6	1218.3
9	1.0	−1	0.4	−1	0.3	−1	1.5	1	0.15	1	0.015	1	1,262.015.3	1229.8
10	1.8	1	0.4	−1	0.3	−1	0.5	−1	0.15	1	0.015	1	371.320.8	328.4
11	1.0	−1	1.0	1	0.3	−1	0.5	−1	0.05	−1	0.015	1	223.69.5	190.5
12	1.0	−1	0.4	−1	0.3	−1	0.5	−1	0.05	−1	0.005	−1	240.08.8	348.1

(1) $$x_{i}=\left(X_{i}-X_{0}\right)/△\,X_{i}$$

where *X*_*i*_ is the coded value of an independent factor, Δ*X*_*i*_ is the step-change in the actual value of the variable *i*, *X*_0_ is the actual value of an independent factor at the center point, and *Xi* is the actual value of an independent factor. The main effect of each salt was calculated following the first-order model equation:

(2) $$x_{i}=2\,\left(\sum {\text{M}}_{\text{i}}^{+1}-{\text{M}}_{\text{i}}^{-1}\right)/N$$

where X*i* is the effect of the tested salt, Mi^+1^ and Mi^–1^ represent the variable response (salt) from the trials, where the salt was measured at a higher and lower concentration, respectively, and *N* is the total number of trials.

#### Optimization and Modeling by Rotatable Central Composite Design

The modeling technique of RSM was employed. For multiple regression analysis, a properly designed experiment was performed to resolve multivariate equations simultaneously. The interaction among the significant salts (NH_4_Cl and KH_2_PO_4_), selected based on the previous PBD trial, in addition to the fermentation period, was optimized. The other salts were omitted from the feather-based medium. Hence, the RCCD of RSM was applied to provide a constant prediction variance at all points that are equidistant from the design center. Hence, the three variables were tested at five levels each. The design matrix, in terms of coded units, composed of the high and low factorial (± 1) levels as well as their center point (_0_) to estimate the pure error, and the design was further augmented with the axial (stars) points located at a specified distance (alpha, α = 1.682) from the design center in each direction on each axis to estimate the curvature (Table 2).

**TABLE 2:** The RCCD matrix of the independent factors, showing the experimental data of amino acids production, and the corresponding predicted values obtained from RCCD and ANN models.

Run	KH_2_PO_4_ (mg/g CFP)		NH_4_Cl (mg/g CFP)		Incubation time (day)		Amino acids (μg/g CFK)				
							Observed	RCCD		ANN	
	Coded (X1)	Actual	Coded (X2)	Actual	Coded (X3)	Actual		Predicted	Residual	Predicted	Residual
1^*^	−1	0.400	−1	1.00	−1	7.00	1,102.30.9	1,127.2	–24.9	1,080.3	22.0
2	1	0.700	−1	1.00	−1	7.00	1,069.42.2	1,035.9	33.5	1,069.0	0.4
3	−1	0.400	1	2.00	−1	7.00	974.91.4	947.2	27.7	980.2	–5.3
4^*^	1	0.700	1	2.00	−1	7.00	637.62.3	658.0	–20.4	647.9	–10.3
5^*^	−1	0.400	−1	1.00	1	14.00	1,111.11.9	1,136.2	–25.1	1,149.1	–38.0
6^*^	1	0.700	−1	1.00	1	14.00	1,098.41.6	1,137.7	–39.3	1,091.6	6.8
7^*^	−1	0.400	1	2.00	1	14.00	1,189.35.1	1,225.5	–36.2	1,189.4	–0.1
8	1	0.700	1	2.00	1	14.00	1,059.94.6	1,029.3	30.6	1,055.6	4.3
9^*^	−1.682	0.298	0	1.50	0	10.50	1,040.41.6	1,025.1	15.3	1,039.9	0.5
10^*^	1.682	0.802	0	1.50	0	10.50	791.02.5	783.3	7.7	762.1	28.9
11	0	0.550	−1.682	0.66	0	10.50	1,285.12.9	1,280.7	4.4	1,298.1	–13.0
12	0	0.550	1.682	2.34	0	10.50	1,019.41.2	1,038.1	–18.7	1,030.5	–11.1
13^*^	0	0.550	0	1.50	−1.682	4.61	961.61.5	989.0	–27.4	994.0	–32.4
14^*^	0	0.550	0	1.50	1.682	16.39	1,312.13.0	1,308.7	3.4	1,337.8	–25.7
15^*^	0	0.550	0	1.50	0	10.50	1,601.52.1	1,626.0	–24.5	1,626.0	–24.5
16	0	0.550	0	1.50	0	10.50	1,541.73.1	1,626.0	–84.3	1,626.0	–84.3
17^*^	0	0.550	0	1.50	0	10.50	1,649.45.1	1,626.0	23.4	1,626.0	23.4
18	0	0.550	0	1.50	0	10.50	1,709.93.6	1,626.0	83.9	1,626.0	83.9
19	0	0.550	0	1.50	0	10.50	1,602.33.1	1,626.0	–23.7	1,626.0	–23.7
20^*^	0	0.550	0	1.50	0	10.50	1,651.53.3	1,626.0	25.5	1,626.0	25.5
21	−1	0.400	−1	1.00	−1	7.00	1,081.82.9	1,127.2	–45.4	1,080.3	1.5
22	1	0.700	−1	1.00	−1	7.00	1,063.42.8	1,035.9	27.5	1,069.0	–5.6
23	−1	0.400	1	2.00	−1	7.00	983.11.3	947.2	35.9	980.2	2.9
24	1	0.700	1	2.00	−1	7.00	647.53.1	658.0	–10.5	647.9	–0.4
25^*^	−1	0.400	−1	1.00	1	14.00	1,181.81.6	1,136.2	45.6	1,149.1	32.7
26^*^	1	0.700	−1	1.00	1	14.00	1,103.11.5	1,137.7	–34.6	1,091.6	11.5
27	−1	0.400	1	2.00	1	14.00	1,190.24.4	1,225.5	–35.3	1,189.4	0.8
28	1	0.700	1	2.00	1	14.00	1,058.73.8	1,029.3	29.4	1,055.6	3.1
29^*^	−1.682	0.298	0	1.50	0	10.50	1,039.12.0	1,025.1	14.0	1,039.9	–0.8
30	1.682	0.802	0	1.50	0	10.50	761.02.6	783.3	–22.3	762.1	–1.1
31	0	0.550	−1.682	0.66	0	10.50	1,308.51.8	1,280.7	27.8	1,298.1	10.4
32	0	0.550	1.682	2.34	0	10.50	1,039.21.1	1,038.1	1.1	1,030.5	8.7
33	0	0.550	0	1.50	−1.682	4.61	997.61.2	989.0	8.6	994.0	3.6
34	0	0.550	0	1.50	1.682	16.39	1,338.92.4	1,308.7	30.2	1,337.8	1.1
35	0	0.550	0	1.50	0	10.50	1,711.61.6	1,626.0	85.6	1,626.0	85.6
36^*^	0	0.550	0	1.50	0	10.50	1,563.23.7	1,626.0	–62.8	1,626.0	–62.8
37	0	0.550	0	1.50	0	10.50	1,549.45.2	1,626.0	–76.6	1,626.0	–76.6
38	0	0.550	0	1.50	0	10.50	1,640.63.2	1,626.0	14.6	1,626.0	14.6
39^*^	0	0.550	0	1.50	0	10.50	1,699.34.1	1,626.0	73.3	1,626.0	73.3
40^*^	0	0.550	0	1.50	0	10.50	1,588.74.5	1,626.0	–37.3	1,626.0	–37.3

*Runs that were selected randomly to serve for validating the model of ANN; the other 22 runs served for model training.

The design matrix was laboratory performed and the AA was determined as the response variable. The experimental data were statistically assessed by using multiple regression analysis, and the analysis of variance (ANOVA) was checked to compare and evaluate the significance of the regression model. The selected polynomial quadratic model was fitted to the next second-order equation:

(3) $$Y=\beta_{0}+\sum \beta_{i}\,X_{i}+\sum \beta_{i\,j}\,X_{i}\,X_{j}+\beta_{i\,i}\,X_{i}^{2}$$

where *y* is the predicted response of AA, β_0_ is the model constant, *X*_*i*_ and *X*_*j*_ are the nutritional salts (independent variables), β*i* is the linear coefficient, β*ij* is the cross-product coefficient, and β*ii* is the quadratic coefficient. This model was laboratory validated based on the statistical analysis of the data.

The predicted response value was calculated based on the preceding equation model and was subjected to laboratory validation to confirm the fitness and accuracy of the theoretically estimated value of each point of the design.

#### Modeling Using Artificial Neural Network

A fully connected neural network platform was constructed with two hidden layers. All nodes within the layers have the hyperbolic tangent sigmoid activation function (NTanH). Response data of the RCCD matrix was used to train the machine and develop the predictive model. The data were portioned into training, testing, and cross-validation sets, in which 22 runs were used randomly, by the software, for training, while the other 18 data points were used for testing and validation. The neural network had one input layer with three nodes (KH_2_PO_4_, NH_4_Cl, and incubation time) and an output layer with one node (AA biosynthesis). Both layers are fixed by the number of the tested independent and response factors, respectively. The in-between layer was constructed and tested using various hidden layers and neurons at various learning rates.

The machine was adjusted to perform a 10,000 tour of learning, applying the trial-and-error search method until the ANN achieved the best performance in the AA prediction, i.e., the highest value of *R*^2^. The trained network performance test was evaluated based on the precision of the neural network to predict outputs that are either similar or very close to the response target value. JMP^®^ Pro software (JMP, Version 14.3) was used.

### Comparison of the Fitness of Rotatable Central Composite Design and Artificial Neural Network Models

The fitness of AA production models generated by RCCD and ANN was assessed and compared, estimating *R*^2^, root mean square error (RMSE), mean absolute deviation (MAD), and the sum of squared errors (SSE). Besides, the values predicted by both models were plotted against the corresponding trial values to explore the fitness of both models.

### Specifications of Amino Acids Using High-Performance Liquid Chromatography

The hydrolysate of the fermented keratinaceous biomass was investigated using an Agilent 1260 series high-performance liquid chromatography (HPLC) for detection of the AA profile (Jajiæ et al., 2013). Briefly, the separation was carried out using the Eclipse Plus C18 column (4.6 mm × 250 mm i.d., 5 μm). The mobile phase consisted of buffer (sodium phosphate dibasic and sodium borate), pH 8.2, and ACN:MeOH:H_2_O 45:45:10 at a flow rate of 1.5 ml/min. The extraction procedure was done by mixing 1.25 ml of the sample with 1.25 ml H_2_O and 2.5 ml of HCl (to a final HCl concentration of 6 M), heated at 100°C for 24 h, and then filtered. Finally, 1 ml of the filtrate was injected into HPLC. Data were presented in μg/g CKF.

### Cell Toxicity Assay

For determination of the cytotoxic agents and any other biologically active compounds that exist in the AA hydrolysate, the hydrolysate was assessed for the cytotoxicity at various concentrations, using 3-(4,5-dimethylthiazol-2-yl)-2,5-diphenyltetrazolium bromide (MTT). Vbrant^®^ MTT cell proliferation assay kit, the standard 96-well microplates, was used.

The cell line of HepG2 was seeded at a density of 5,000 cells per well with 10 μl of 12 mM MTT, including a negative control of only 100 μl of medium alone, then incubated at 37°C for 4 h in a humidified chamber. The MTT assay involves the conversion of the water-soluble MTT to an insoluble formazan, which is then solubilized, and its concentration was determined by optical density at 570 nm, using DMSS (Mosmann, 1983).

### Trial Design and Statistical Examination

The screening matrix of PBD, the design matrix of RCCD, the regression analysis, and ANOVA were operated using the statistical software package Minitab software (version 19.2, Minitab Inc., United States). The modeling and the topology of ANN were operated using JMP Pro software (JMP^®^, Version 14.3, SAS Institute Inc., Cary, NC, 1989–2019), which enables training, validating, and testing using experimental data with several hidden layers and neurons. To enhance the prediction accuracy of both RCCD and ANN models, experiments of RCCD were repeated twice. All trials were implemented in triplicate, and the significance threshold was set at probability (*P*) level ≤0.05. The mean of each experimental run was introduced ± standard deviation (SD).

## Results

### Selection of Keratinolytic Bacterium

**FIGURE 1:**
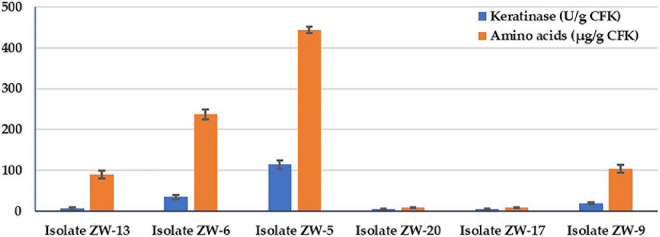
Bacterial screening for keratinase activity and total amino acids produced during fermentation on CFK.

The six isolated keratinolytic bacteria were picked up based on their ability to grow on keratin agar medium. The bacterial isolates were quantitively screened for the keratinase activity and AA production on the SSF medium (Figure 1). Data illustrate that the bacterial isolate ZW-5 had the highest activity in keratinase activity and total AA produced during the CFK fermentation process. The keratinase activity of the bacterial isolates in descending order were 113.8, 34.2, 19.4, 6.2, 5, and 5 KA (U/g CFK) for isolates ZW-5, ZW-6, ZW-9, ZW-13, ZW-17, and ZW-20, respectively. The AA production pattern of bacterial isolates in descending order were 444.3, 236.9, 103.9, 89.4, 9, and 9 μg/g CFK for isolates ZW-5, ZW-6, ZW-9, ZW-13, ZW-17, and ZW-20. Data of the simple correlation coefficient (*r*) between KA and AA was calculated and was found to be significant (*r* = 0.967). The bacterial isolate ZW-5 is a promising keratinolytic isolate and, therefore, was chosen for further studies on AA production.

### Identification of Selected Isolate

**FIGURE 2:**
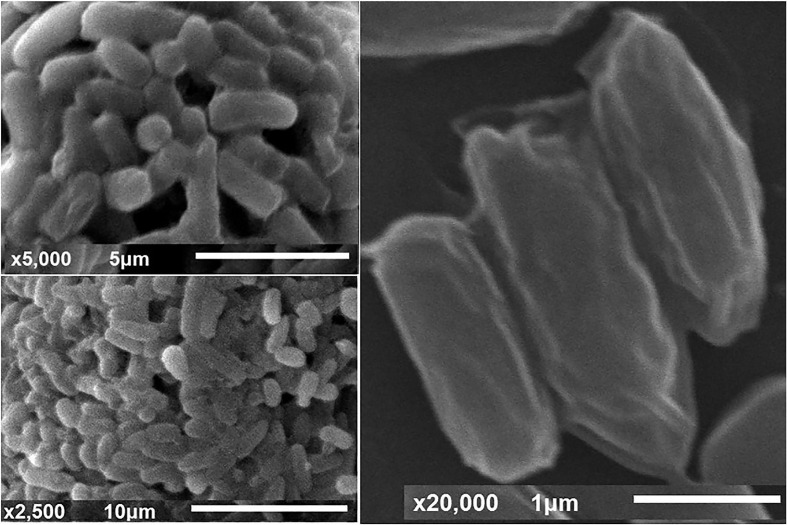
SEM micrograph of *Bacillus paramycoides* at various magnification powers.

The cell’s morphological features and biochemical tests showed that the bacterial isolate is gram-positive, non-motile, and facultatively anaerobic. As illustrated by SEM (Figure 2) the cells have a rod shape with dimensions of 1.1–1.3 μm in width and 2.1–3.0 μm in length. From the morphological, physiological, and biochemical features, the bacterium ZW-5, therefore, is proposed to be included in the genus *Bacillus* as a representative of a novel sub-species, *Bacillus paramycoides*.

**FIGURE 3:**
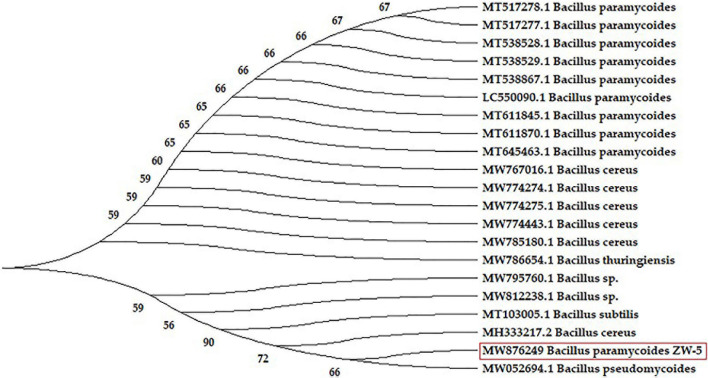
Neighbor-joining phylogenetic tree based on 16S rRNA sequences, showing the relationships between strain ZW-5 (inside the red rectangle), and related species of *Bacillus* spp. GenBank accession numbers are presented in the front of strain names.

Molecular phylogeny was constructed. The evolutionary relationships were inferred using the Neighbor-Joining method. The bootstrap consensus tree inferred from 1,000 replicates to represent the evolutionary history of the analyzed bacterial sequences (Figure 3). The evolutionary distances were computed using the Jukes–Cantor method. The sequence analysis of the current strain (ZW-5) was compared with 20 16S rRNA nucleotide sequences with about 99% similarity to the strain ZW-5. The strain was aligned with the representative *Bacillus* spp. retrieved from the GenBank databases by using nBLAST searches. The phylogenetic analysis indicated that strain ZW-5 formed a distinct branch with five other *Bacillus* spp. The strain ZW-5 was identified as *B. paramycoides* with the GenBank accession number MW876249.

### Screening of Medium Components Using Plackett–Burman

Assuming that the salts’ coefficients are equal to zero, implying that there is no association between the salts and the AA production (null hypothesis), a combination of mineral salt solution was investigated, based on the matrix of PBD, to estimate their significance and relative importance on AA production (Table 1). The results show obvious variation among the various runs, of AA production, yet, the predicted values of the AA are relatively close to the experimental values.

**FIGURE 4:**
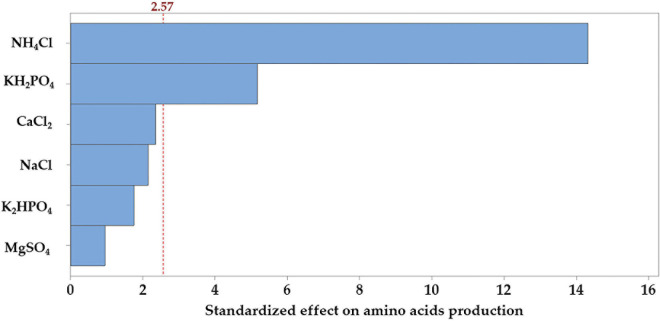
Pareto chart, displaying the standardized effects of each of the tested salts on the production of the amino acids by *B. paramycoides* ZW-5.

The Pareto chart of the standardized effects was generated to determine which of the tested salts contribute the most to the variability in the response (Figure 4). The relative magnitude and the absolute value of the standardized effects of the tested salts were figured in descending order. Among the tested salts, NH_4_Cl extends the farthest, recording the most significant effect. On the other hand, the effect of MgSO_4_ on AA production is the smallest one. However, the reference line (2.57) on the chart indicates which effects are significant; in this connection, NH_4_Cl and KH_2_PO_4_ reached the threshold of significance (*p* ≤ 0.05), but the other salts did not reach the level of significance.

For further assessment of the null hypothesis, data (Supplementary Table 1) display the significant impact of the tested salts based on a significance level of *p* ≤ 0.05. The estimated effect and the regression coefficient for the tested salts varied from positive to negative effect. The ANOVA showed that the model term is statistically significant (*P* < 0.001); so, the association between the AA and each salt in the design was compared with the corresponding *p*-value of each term. Salt associated with AA at *p* ≤ 0.05 is statistically significant. Again, NH_4_Cl (*p* < 0.001) followed by KH_2_PO_4_ (*p* = 0.001) has a statistically significant association with AA, both contributing with 81.7% and 10.6% in the model, respectively. The other terms have *p*-values greater than the significance level, concluding that there is no statistically significant association between the AA and CaCl_2_, NaCl, K_2_HPO_4_, or MgSO_4_. However, as indicated by the estimated effect and regression coefficient, NH_4_Cl has a positive significant effect, whereas KH_2_PO_4_ has a negative significant one, meaning that it will best fit at the lower concentration.

Several fitting statistics are usually used for measuring the aptness of the model data. In which the standard deviation was estimated to be 97.11. The values of the different *R*^2^ (coefficient of determination) kinds show reasonably high values, being 98.01 (*R*^2^), 95.62 (adjusted-*R*^2^), and 88.50% (predicted-*R*^2^). Accordingly, the null hypothesis was rejected and NH_4_Cl and KH_2_PO_4_ showed a significant effect; both were subjected to further optimization studies, omitting the other salts from the fermentation medium.

**FIGURE 5:**
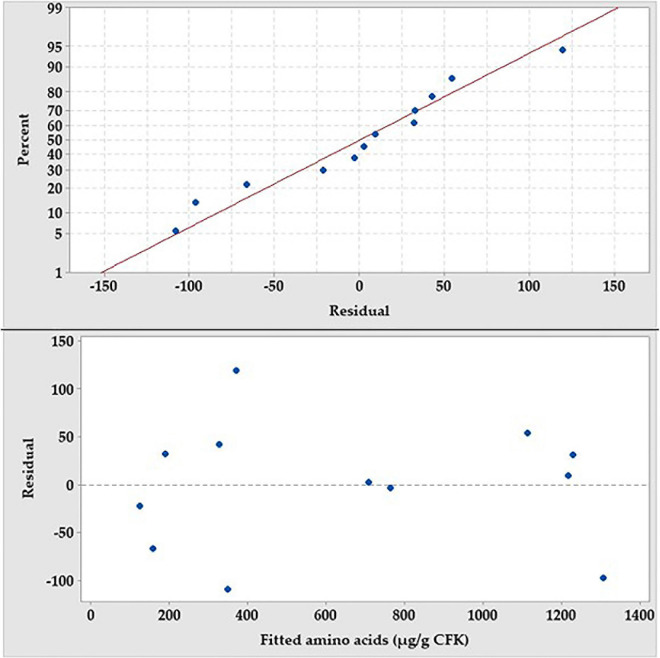
The normal probability plot of the PB residuals and the residual versus fit plot for the production amino acids by *B. paramycoides* ZW-5.

The assumptions of the analysis of PBD were checked, employing the residual analysis. Plotting the normal probability plot of the residuals (Figure 5) shows that the residuals are normally distributed and follow, approximately, a straight line. Also, the residual versus fits plot indicates that the residual points are scattered independently and randomly distributed on both sides of 0, with no recognizable patterns. For simplicity and economic issues, and according to the preceding screening, NH_4_Cl and KH_2_PO_4_ were chosen and the other salts were omitted from the fermentation medium.

### Modeling the Optimization of Amino Acids Assemble by Rotatable Central Composite Design

Optimization of the maximization process of AA production was investigated using the two significant nutritional parameters selected from the preceding PBD, i.e., KH_2_PO_4_ and NH_4_Cl, in addition to the time course profile of the bioconversion process. Hence, the RCCD matrix of three variables at five levels each was established. The other insignificant variables of the medium components were omitted. The design matrix of coded and actual levels of the three variables is presented in Table 2. The observed (measured) AA response, by the keratin-degrading bacterium, against the various runs of RCCD shows considerable variation, ranging from 637.6 (run 4) to 1,711.6 (run 35) μg/g CFK. The predicted values (calculated based on the regression analysis) of the 40 runs were relatively close to the experimentally observed ones. Consequently, the residuals (the difference between the observed and the predicted values) recorded lower values.

#### Regression Coefficient and Analysis of Variance

To assess (accept or reject) the null hypothesis, which implies that the term’s coefficient is equal to zero, and to detect and measure, if any, the statistically significant association between the three tested terms and the AA response, the statistical analysis of the data (Table 3) was performed. The coefficient for the general model as well as the individual, squared, and interaction among the three terms are statistically significant at *p* ≤ 0.05 level, recording various positive and negative coefficients. Thus, the null hypothesis was rejected. The goodness-of-fit statistics were estimated. The standard deviation value (45.27) was relatively low, whereas the values of *R*^2^ (98.86), adjusted-*R*^2^ (97.82), and predicted-*R*^2^ (97.41) were all in the high range. The predicted residual error sum of squares (PRESS) resulted in a value of 94,688.50.

**TABLE 3:** The statistical analysis of the RCCD data of amino acids, generated by *B. paramycoides* ZW-5, shows the coded regression coefficient and ANOVA.

Source	Regression coefficient	Freedom degree	Sum of square	Mean square	*F*-value	*p*-value
Model	1626.0	9	3,596,326	399,592	195.01	<0.001^∗^
**Linear**		3	529,964	176,655	86.21	<0.001^∗^
KH_2_PO_4_	−71.9	1	141,171	141,171	68.90	<0.001^∗^
NH_4_Cl	−72.1	1	142,066	142,066	69.33	<0.001^∗^
Time	95.0	1	246,727	246,727	120.41	<0.001^∗^
Square		3	2,946,040	982,013	479.25	<0.001^∗^
(KH_2_PO_4_)^2^	−255.2	1	1,876,924	1,876,924	916.00	<0.001^∗^
(NH_4_Cl)^2^	−165.0	1	784,391	784,391	382.81	<0.001^∗^
(Time)^2^	−168.7	1	820,091	820,091	400.23	<0.001^∗^
**Two-way interaction**		3	120,322	40,107	19.57	<0.001^∗^
KH_2_PO_4_ × NH_4_Cl	−49.4	1	39,115	39,115	19.09	<0.001^∗^
KH_2_PO_4_ × Time	23.2	1	8,644	8,644	4.22	0.049^∗^
NH_4_Cl × Time	67.3	1	72,563	72,563	35.41	<0.001^∗^
**Error**		30	61,472	2,049		
Lack-of-Fit	–	5	16,101	3,220	1.77	0.155^ns^
Pure Error	–	25	45,371	1,815	–	–
**Total**		39	3,657,797	–	–	–

**The goodness-of-fit statistics of the model**						

Standard deviation					45.27	
Coefficient of determination (*R*^2^)					98.32	
Adjusted-*R*^2^					97.82	
Predicted-*R*^2^					97.41	
Predicted residual error sum of squares					94,688.50	

*Significant term, ^*ns*^ insignificant term.

#### Model Equation and Validation

The statistical analysis revealed very good evidence for the tight relationship between AA production and the independent variables (KH_2_PO_4_, NH_4_Cl, and incubation time). Therefore, the optimum levels of the independent variables were calculated using the second-order polynomial equation that defines predicted AA response in uncoded units:

AA (μg/g CFK) = −4,295 + 12,520 × (KH_2_PO_4_) + 1,794 × (NH_4_Cl) + 234.2 × (incubation time) – 11,342 × (KH_2_PO_4_)2 – 659.9 × (NH_4_Cl)2 – 13.770 × (incubation time)2 – 659 × (KH_2_PO_4_) × (NH_4_Cl) + 44.3 × (KH_2_PO_4_) × (incubation time) + 38.48 × (NH_4_Cl) × (incubation time).

**FIGURE 6:**
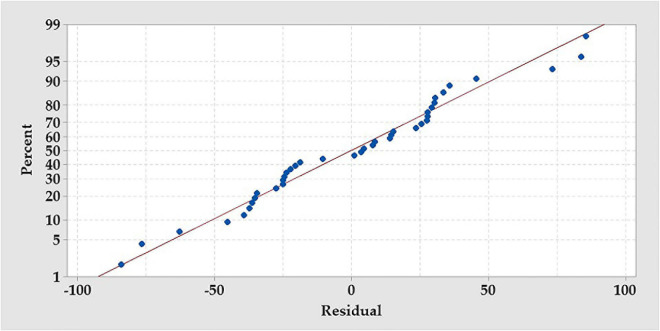
The normal probability plot of the RCCD residuals of the production of the amino acids by *B. paramycoides* ZW-5.

The normal probability plot of the residuals was figured to test their normality (Figure 6). Expect some scatter points, there are no definite patterns, like curves, that could be observed. On the other hand, the residual points were tightly clustered along the standard line.

**FIGURE 7:**
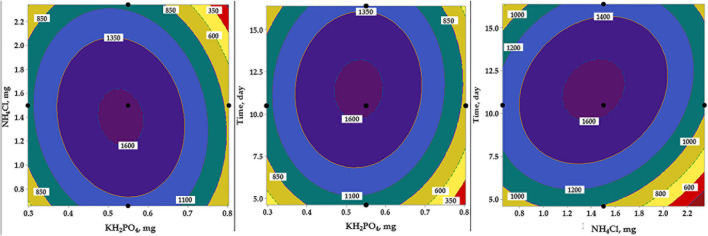
Contour plots show the interactive effects of each pair of the independent variables on amino acids production by *B. paramycoides* ZW-5, holding the third variable at the center point. Hold values are 0.55 mg for KH_2_PO_4_, 1.5 mg for NH_4_Cl, and 10.5 days for incubation time.

The contour plots were generated to visualize and explore the relationship between AA and the mutual interactions among the tested variables (Figure 7). The contour plot surface plot depicts the relation of each pair of factors to AA, which is an aid to understand both the linear and the interaction effects of the two tested variables. The highest amount of AA production after modeling was found to be within the range of each of the tested independent variables proposed in the design matrix, i.e., near to the center point level of the factor.

### Modeling Amino Acids Assemble by Artificial Neural Network

The response data (Table 2) of the RCCD was used for machine learning and developing the predictive ANN model. For such purpose, a fully connected neural network platform with multilayer feed-forward ANN architecture was constructed for modeling the AA production by the selected bacterium. To determine the best architectural structure and the best number of neurons in the hidden layer, numerous hidden neurons, and various combinations of ANN-specific parameters, such as learning rate, were tested. All nodes within the hidden layer have the same activation function, NTanH.

**FIGURE 8:**
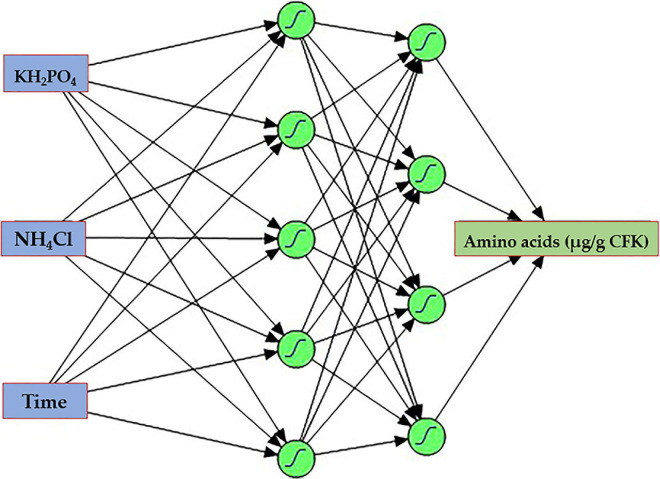
The architecture scheme of the artificial neural network, NTanH(4)NTanH(5), containing one input layer (three neurons), two hidden layers (four and five neurons), and an output layer (one neuron).

The ANN training was performed, employing the holdback procedure at a proportion of 0.45. During several learning trials, each of 10,000 tours, two hidden layers with four and five neurons, and a learning rate of 0.1, using the squared penalty method was the best model that maximizes the AA. The ANN topology (Figure 8) was constructed and designated as 3-5-4-1. The input layer is composed of three neurons (KH_2_PO_4_, NH_4_Cl, and incubation time), representing the number of the tested independent factors. The output layer has one neuron (AA), representing the response factor. The in-between hidden layers performed better when using two hidden layers, the first, NTanH(4), has four, and the second, NTanH(5), has five neurons, designated as NTanH(4)NTanH(5).

The machine learning and validation processes were performed on the constructed ANN with a trial-and-error procedure until the *R*^2^ reached its maximum, which is accompanied by the ANN’s ability to predict outputs that are either similar or very close to the experimental response value. After 10,000 tours, the ANN predicted values of each resulted experimental point were calculated and given along with the predicted RCCD values in Table 2. Consequently, ANN predicted values and their errors showed reasonable agreement with the experimental ones, and more showed lower residual values than those obtained by the RCCD model.

### Comparison of Rotatable Central Composite Design and Artificial Neural Network Models

The validation of the overall model performance by both RCCD and ANN was compared based on the model’s ability to predict AA production accurately. The statistical parameters that measure and compare the accuracy of both models were estimated (Table 4). *R*^2^, RMSE, and MAD statistics were estimated for both training and validation sets of RCCD and ANN. Moreover, the values of *R*^2^ were high for the training and validation processes of the ANN model, compared to the RCCD model. In contrast, RMSE and MAD recorded lower values. The overall performance of both models was evaluated, and the same trend was observed for all the tested statistics, including higher *R*^2^ value and lower RMSE, MAD, and the sum of squares due to error (SSE) of ANN compared to RCCD, concluding that the ANN model is slightly better at the overall classification statistics.

**TABLE 4:** Statistics of the model performance created by RCCD and ANN.

**Training statistics**				
Model	*R* **^2^**	RMSE	MAD	Number of used runs
RCCD	0.9794	42.588	34.444	22
ANN	0.9852	36.122	20.09	22

**Validation statistics**				

RCCD	0.9874	34.617	30.051	18
ANN	0.9893	31.915	25.357	18

**Overall model performance**				

Statistics		RCCD	ANN	Number of used runs
R^2^		0.9832	0.9871	40
RMSE		39.202	34.293	40
MAD		32.467	22.46	40
SSE		61,450.09	47,040.39	40

*Root mean squared error (RMSE), mean absolute deviation (MAD), the sum of squares error (SSE).*

**FIGURE 9:**
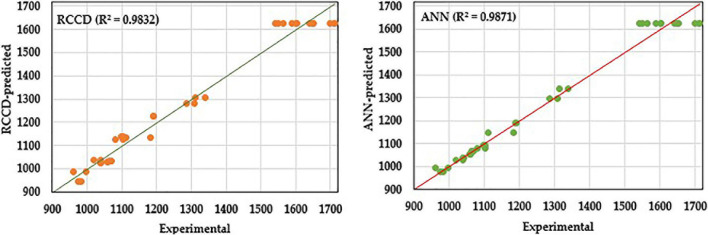
Experimental values against predicted values by RCCD or ANN model for amino acids biosynthesis by *B. paramycoides* ZW-5.

Likewise, the predicted values by both models were plotted against the corresponding actual (experimental) values to compare the fitness of both models (Figure 9). ANN proved, again, its higher predictive capacity than the RCCD model, in which the linear regression analysis displays better fitting by the ANN model prediction points that lie much closer to the line of perfect prediction than the RCCD model.

### Laboratory Validation of Both Models

To determine the model’s prediction accuracy and verify the optimization results, the optimal predicted (estimated) levels of the tested factors were estimated by both models (RCCD and the well-learned ANN) and then laboratory validated; afterward, the true value was compared to the calculated theoretical AA value to check the predictive proficiency of both models. The theoretical levels of the three variables were calculated to be 0.547 mg KH_2_PO_4_, 1.438 mg NH_4_Cl, and 11.61 days of incubation, which were expected to yield 1,627.47 and 1,636.29 μg AA/g CFK by RCCD and ANN models, respectively. These levels of variables and their response were validated under the laboratory to check and compare the models’ applicability. The experimental value was found to be 1,634.43 ± 1.56 μg/g CFK. Generally, the value corresponds very well to the theoretically predicted value, substantiating the suitability of the developed models. However, the experimental validation value is more obeyed and closer to that estimated by the ANN model than RCCD.

### Screening the Amino Acids Profile

**FIGURE 10:**
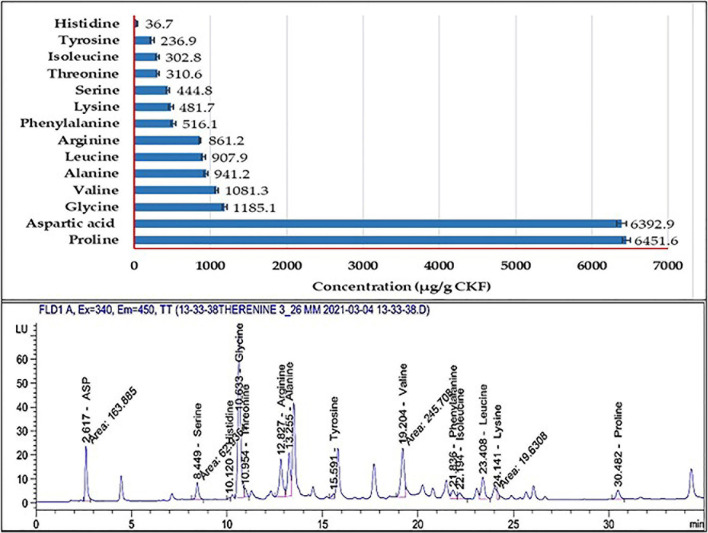
HPLC analysis of the amino acids’ profiles released from the fermented feather by *B. paramycoides* ZW-5.

Results of HPLC (Figure 10) show 14 AA released from poultry feathers degraded by *B. paramycoides* ZW-5 with a total concentration of 20,150.1 μg/g CKF. Due to CKF fermentation, seven essential AA were released (histidine, threonine, valine, phenylalanine, isoleucine, leucine, and lysine). While seven non-essential AA (aspartic acid, serine, glycine, arginine, alanine, tyrosine, and proline) were released. Four of them (glycine, arginine, tyrosine, and proline) are conditionally essential. The highest concentrations were recorded with proline (6,451.6 μg/g CKF) and aspartic acid (6,392.9 μg/g CKF), followed by glycine (1,185.1), valine (1,081.3), alanine (941.2), and leucine (907.9 μg/g CKF).

### Investigation of the Cell Toxicity of the Crude Amino Acids

**FIGURE 11:**
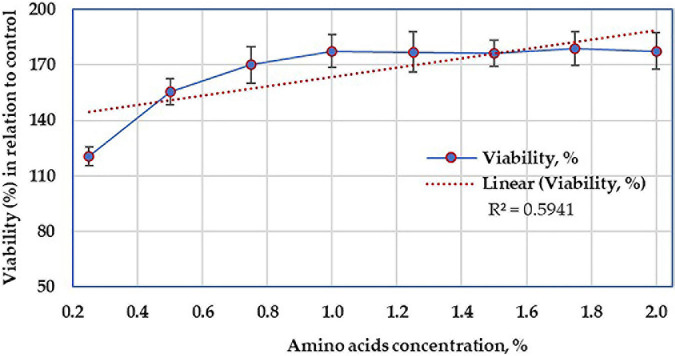
Cytotoxicity test showing the viability of the mammalian cell line in the presence of various concentrations of amino acids.

The AA-treated and control HepG2 cells demonstrated various viability reactions (Figure 11) that varied according to the concentration of AA. However, the viability of cells increased with the higher concentrations of AA, up to 1%, then constant up to 2%. Anyhow, this linear relationship was not significant (*R*^2^ = 0.5941). Moreover, there was no morphological alteration in HepG2 cells.

## Discussion

Avian feather is limitedly utilized as a source of nitrogen for plants or as a dietary protein supplement for animal feedstuffs. For that purpose, feathers are chemically treated to increase the digestibility and reduce rigidity, leading to the degradation of certain heat-sensitive AA, like methionine, lysine, and tryptophan, and may generate non-nutritive AA, such as lysino-alanine and lanthionine. That is why enzymatic biodegradation is a better alternative to improve the nutritional value and offers cheap and mild reaction conditions for the biodegradation process (Marcondes et al., 2008; Saber et al., 2010; Nnolim et al., 2020). So, keratin-degrading enzymes offer a feasible microbial technology for obtaining keratinolytic enzymes.

An account of bacterial and fungal species showing the potentials for the degradation of avian feathers abound. Most fungi with excellent keratinolytic properties have been reported to exist among the dermatophytes, which creates limitations for useful application. Therefore, finding out alternative keratinolytic bacteria represents an advantage over the fungal. The bio-catalytic efficiency of the keratinases produced by bacteria species tends toward a broad spectrum of proteinaceous substrates and is robust at extreme conditions (Saber et al., 2010; Nnolim et al., 2020).

Regarding bacterial selection (Figure 1), there was a significant correlation coefficient (*r* = 0.967) between KA and AA. This is because keratinase enzymes are in charge of driving such a process. The feathers’ keratin is an insoluble protein resistant to degradation by common proteolytic enzymes because of a high degree of cross-linking by disulfide bonds, hydrogen bonding, and hydrophobic interactions (Marcondes et al., 2008).

Keratin is an insoluble protein resistant to degradation by common proteolytic enzymes. Microbial keratinases (EC 3.4.21/24/99.11) are special proteolytic enzymes that can break down the insoluble keratin such as feather into peptides and/or AA in a simple way. The enzymes are also characterized by their high substrate specificity to keratin (Saber et al., 2010; Tamreihao et al., 2019).

After selection of the most potent keratinolytic bacterium, it was fully identified. The features of the selected bacterial isolate were found to be compatible with the ideal characters of *B. paramycoides* that were previously described (Liu et al., 2017). Moreover, all identification procedures, including TEM (Figure 2), molecular phylogenetic analysis (Figure 3), and morphological and biochemical ones, came into agreement that the bacterial isolate is *B. paramycoides*, representing the first record of such species as keratinolytic bacterium since it was first reported as a novel member of the *Bacillus cereus* group (Liu et al., 2017). To the best of the authors’ knowledge, the current bacterial species is the first reported keratinolytic one.

Generally, keratin-degrading efficiency is well reported in several bacteria, especially *Bacillus* spp., that can thrive on the keratinous biomass as the sole source of nitrogen, and carbon, indicating the secretion of protein- and keratin-degrading enzymes (Nnolim et al., 2020; Bhari et al., 2021; Cavello et al., 2021; Li, 2021). *Bacillus* spp. has been known as the predominant bacteria group that mediates the degradation of keratin compounds through keratinases and, moreover, has gained much attention for keratinase production because of scale-up feasibility, easy handling, and high commercial applicability (Nnolim et al., 2020). The species include *B. subtilis, B. aerius, B. pumilus, B. cytotoxicus*, *B. cereus*, and *B. lichenifomis* (Cavello et al., 2021; Li, 2021). The latter is the first commercial keratinase preparation source (Bhari et al., 2021). The current *B. paramycoides* ZW-5 is another new candidate in this respect.

The bacterial nutritional requirements for the degradation of the avian feathers were monitored using a PBD matrix (Table 1). It is well known that PBD is used to accomplish two major goals: the first is to determine which of the tested factors are important (significant); consequently, the second is to define which level (high or low) of the significant factor(s) should be used when doing further optimization studies.

The Pareto chart (Figure 4) analysis was applied to determine the absolute values of the standardized effects from the largest to the smallest one and to indicate which effects are statistically significant, as well. Consequently, NH_4_Cl and KH_2_PO_4_ are statistically significant with the current model terms. However, the Pareto chart can determine which effects are large and significant, but cannot determine the direction of the effects (low or high) that can increase the response (AA). Therefore, coefficients and ANOVA analysis were used to examine the magnitude and determine the direction of the effects. If the estimated effect of the regression coefficient for a variable (salt) is negative, a lower concentration is to be required during further optimization studies, and vice versa. The changes varied from positive (NH_4_Cl) to negative (KH_2_PO_4_) effect.

The significance of the model (at *p* ≤ 0.05) (Supplementary Table 1) indicates that changes in the value of the variables (salts) are associated with changes in the mean response value (AA). A significance at *p* ≤ 0.05 indicates a 5% risk of concluding the presence of an association between salts and AA when, actually, there is no association (Elsayed et al., 2021). The same role is applied for each of every individual variable. According to the coefficients and *p*-value, it could be concluded that changes in NH_4_Cl (at the higher level) and KH_2_PO_4_ (at a lower level) are significantly associated with changes in AA at the significance level of 0.05.

To decide the fitness of the model, the goodness-of-fit statistics were estimated. Of them, the standard deviation recorded a low value (97.1) since this statistic is measured in the units of the response variable (AA), representing how far the data values fall from the fitted (predicted) ones. The lower the value of standard deviation, the better the model describes the response. However, a low standard deviation value by itself does not indicate that the model meets the model assumptions, so the residual plots should be checked to verify the assumptions.

The value of *R*^2^ and adjusted-*R*^2^ are other goodness-of-fit statistics, and both recorded high values. The value of *R*^2^ defines the variation quantity in the experiential response values that are described by the factor(s). Adding items to the model leads to get bigger *R*^2^, but adjusted-*R*^2^ is not because it depends on the significance of the factors, not their number, in the model. However, the higher the adjusted-*R*^2^, the additional accuracy of the relationship between the factors and the response (AA); consequently, the model fits well the data. Predicted-*R*^2^ illustrates how well the model predicts the responses in the new experiments without over-fitting. Greater predicted-*R*^2^ value suggests a high prediction efficiency of the model. The current model explains 98.0% of the variation in AA, indicating that the model provides a good fit to the data with a prediction accuracy of 88.5%.

Residual analysis was performed (Figure 5) to check whether the PBD model meets the assumptions of the analysis. Residuals are the differences between the experimental (observed) value of the dependent variable (AA) and its corresponding predicted value at each data point. The lower the value of the residuals, the more fit the model data, which consequently signifies the accuracy of the parameter selection. Depicting the normal probability plot of the residuals shows a straight line. Patterns, other than a straight line, indicate that the model does not meet the model assumptions (Saber et al., 2021). Unusual patterns that show a non-straight line, a point that is far away from the line, or changing slope indicate non-normality, an outlier, or an unidentified variable, respectively. Moreover, plotting the residual versus fit shows that the points are distributed, randomly, around the centerline, without recognizable patterns, indicating that they have constant variance and there is a correlation between any of the residual points. The only one inferring could be obtained from both residual tests; the model is adequate, enough, to meet the assumptions of the analysis and fits the data without any caution when interpreting the results.

The design matrix of RCCD (Table 2) was selected to study the interaction between NH_4_Cl and KH_2_PO_4_, which were the chosen salts to be added to the fermentation medium, and the other salts were omitted. Such composition represents an easy-to-prepare medium, besides being lower in cost from an economical point of view. Incubation time was also incorporated to determine the optimum fermentation period.

The multiple regression analysis was performed on the data recovered by RCCD. In regression analysis, the difference between the experimental and the calculated predicted values, at each data point, is the residual (Table 2), which has a vital role in validating the regression model; it can determine the appropriateness of the generated model as a linear or non-linear regression. The lower the value of the residuals, the more fit the model data, which consequently signifies the accuracy of the parameter selection and further indicates that the variance is independent of AA production, thus supporting the adequacy of the model fit.

According to the regression coefficient and ANOVA tests (Table 3), and the previous discussion on the sense of *p*-value, the null hypothesis was rejected to the general model and all its terms. Consequently, the alternative hypothesis was accepted, since the term’s coefficient is not equal to zero, meaning that there were significant differences for all the three tested parameters and their interaction. The *p*-value at ≤0.05 was used as a tool to check the significance of the coefficient of the model and every single factor, which, in turn, is necessary to understand the pattern of the mutual interactions between the test variables.

The significant coefficient of the individual factor indicates that not all level means are equal, whereas a squared term refers that the relationship between the factor and the response follows a curved line. The significant coefficient for an interaction term means that the relationship between a factor and the response depends on the other factor(s) in the term, and the interaction effect must be considered when interpreting the main effects.

Furthermore, the pattern of the coefficients is interpreted based on the signs (positive or negative effect on the response), whereas the interaction between two factors could appear as an antagonistic (negative coefficient) or a synergistic effect (positive coefficient). Inferring from the above, the model implies a significant association between AA and the all-tested terms. They are limiting factors in the model, and little variation in their values strictly alters the AA production rate. Interestingly, none of the terms recorded a greater *p*-value than the significance level, so there was no need to reduce the model terms by refitting without the insignificant term(s).

A collection of statistics was estimated to determine how well the model fits the data. Of which, *R*^2^ of 98.86 indicates that the response variations are attributed to the independent variables, and only just 1.68% of the total variations cannot be explained by the model. A regression model with *R*^2^ ≥90 reflects a perfect fit between the observed and predicted values (Chen et al., 2009; El-Hersh et al., 2017). This role is in line with the current data and could be applied to the other two *R*^2^ kinds; the adjusted-*R*^2^ and predicted-*R*^2^. This implies that the model is reliable for AA production with the tested factors in the present study. Additionally, the high value of predicted-*R*^2^ indicates how well the model predicts responses for new observations that are not tested in the design. PRESS is a critical output in regression analysis; a smaller PRESS indicates minor variation in the data with better modeling. PRESS statistic is a form of cross-validation used in regression analysis, together with the predicted-*R*^2^, to measure the fit of a model to observations that were not themselves used to estimate the model, thus improving the predictive ability of the model.

Residual normality and contour plots were generated to evaluate and explore the RCCD model. Plotting the residual’s normality (Figure 6) displayed no definite patterns, and various residual points of the design matrix of RCCD were tightly clustered along the standard line, indicating that the residuals follow the normal distribution (Saber et al., 2021). The two-factor interaction represented by the contour plots in relation to AA production (Figure 7) was used to understand both the linear and the interaction effects of the two tested variables, as well as to calculate the optimal level of each factor that maximizes the response. The contour plots display a clear interaction between the tested parameters. However, the peak production of AA was located around nearly the center points of the design, reflecting the precision of the selected range of both tested factors (Saber et al., 2021).

Regarding inorganic salts, nitrogen and phosphorus salts were found to have a pronounced effect on the biodegradation of keratin. However, the preference of a nitrogen source depends on the microbial strain used for fermentation conditions. NH_4_Cl was the best inorganic nitrogen source for AA production by the candidate bacterium. This may be back to its simple structure, which does not require a complicated biological metabolism for its assimilation (El-Metwally et al., 2016; Negm et al., 2021). The same could be applied to KH_2_PO_4_, which represents an initial source of P during the early stage of fermentation since inorganic phosphorus positively affects microbial growth (Miettinen et al., 1997). In contrast to the present study, neither ammonia sulfate nor sodium nitrate at any concentrations exerted a positive effect on keratinase production, while K_2_HPO_4_ was found to stimulate the degradation process by *B. licheniformis* (Ni et al., 2011).

Following the fermentation conditions reported in the current study, marked 3.73-fold maximization of the AA production by the selected *B. paramycoides* ZW-5 was gained; this, in turn, reflects the efficacy and accuracy of the statistical optimization procedure. Furthermore, the peak production of AA was found within the range of the design points of each factor, implying the exactness of the selected range of the factors.

Modeling with ANN was another optimization procedure. Given the comparison statistics, both RCCD and ANN models exhibited high predictive ability. Comparing both models (Table 4) reveals that the ANN model has a significantly higher generalization capacity than the RCCD model. ANN models were better than RCCD, recording lower RMSE, MAD, SSE, and *R*^2^. The modeling ability of a given model is reliant on the high value of *R*^2^ and lower values of the RMSE, MAD, and SSE. *R*^2^ measures the correlation between the response values and the predicted values, so a higher value (up to 1) reflects a strong correlation between both datasets. Commonly, RMSE is used in regression analysis to authenticate experimental results since the lower value means that the data are concentrated around the line of best fit (prediction errors). MAD is another statistic that determines the average dispersion of the data around the mean, and a lower value indicates a lower spread of the data around the mean. Finally, SSE, another assessment of the goodness-of-fit, determines the total deviation of the response values from their fitted values, and a lower value implies more fitness of the model. Therefore, ANN has consistently performed better than the RCCD in all aspects. ANN has a universal ability to approximate the nonlinearity of the system, compared with the restricted nature of RCCD to the second-order polynomial, which requires only a sole step calculation for a response surface model (Ram Talib et al., 2019; Saber et al., 2021). Another overall comparison was performed, in which the linear regression analysis between the experimental values and those predicted ones was figured (Figure 9). The ANN model prediction points lie much closer to the line of perfect prediction than the RCCD model. Thus, the ANN model has a significantly higher generalization capacity than the RCCD model.

Although ANN was better than RCCD, the latter still has some merits; i.e., the structured nature of the RCCD can demonstrate the contributions of each factor in the regression models, thus recognizing the insignificant factors, whether single, interaction, or quadratic, in the model and thereby can be eliminated from the model. Moreover, compared to RCCD, ANN modeling consumed extended computational time through many iterative calculations (Saber et al., 2021).

Following the fermentation conditions reported by both models, the theoretically predicted value of AA was calculated and validated in comparison to the experimental one to compare the predictive capability of both models. Applying the calculated theoretical values of the three tested variables under laboratory conditions was found to be more closely related to the speculative value of ANN than RCCD. Such a result truly confirms the higher accuracy and predictive ability of the ANN model than the RCCD one.

Notably, the modeling process results in a total of 1,634.43 ± 1.56 μg/g CFK, representing a 3.678-fold increment in AA production compared with that obtained by *B. paramycoides* ZW-5 at the initial screening trial (444.3 μg/g CFK). This, basically, declares the importance and recommends the application of ANN in the modeling during the avian keratin biodegradation process.

Under the optimum conditions reported above, the AA profile in the hydrolysate of the fermented feather was analyzed. The HPLC analysis (Figure 10) showed the presence of 14 AA distributed between essential and non-essential ones. This, in turn, indicates the success of both fermentation techniques by the new candidate bacterium, from one side, and the sensation of the modeling system applied during the fermentation on the other side. However, the type and amount of the released AA differ according to the substrate, microbe, and condition of fermentation. For instance, the biodegradation of chicken feather waste by *B. aerius* NSMk2 released 17 free AA, where eight were essential (Bhari et al., 2020). *B. cereus* PCM 2849 was able to release both essential and non-essential AA where the glutamic acid and proline were found at the highest concentrations (Ciurko et al., 2019). *Bacillus* sp. UPM-AAG1 recorded the highest concentration of phenylalanine (Abdul Gafar et al., 2020).

Proline and aspartic acid were the major AA in the fermented hydrolysate of CFK. Proline has a multifunctional role in plants and animals. Under plant stress, it maintains the redox state, enhances chlorophyll content, stabilizes protein structure, detoxifies heavy metals, activates DNA-repairing enzymes, and osmoregulates compounds (Siddique et al., 2018). Aspartic acid plays a notable role in the resistance of the plant to salt stress by increasing cell membrane tolerance and enhancing gene expression of salt exclusion. It also enhances seed germination and the vigor of seedlings (Zhang et al., 2020).

In animals, proline is a regulatory axis in cancer and metabolism-dependent models, that is, senescence, and development of embryonic stem cells (Phang, 2019). Aspartic acid plays an important role in vertebrate reproduction, through enhancing the testosterone levels in male animals and improving reproductive activity and semen quality, proposing therapeutic applications of this AA in andrology (Di Fiore et al., 2019).

The growth of bacteria is known to be accompanied by the secretion of some secondary metabolites, other than AA. The cytotoxicity was performed to ensure that the crude AA hydrolysate does not contain any other bacterial secondary metabolites, having an adverse effect on living cells; what is more, the optimum AA concentration was also addressed at 1%. Regarding cell toxicity (Figure 11), the determination of cell growth rates is widely used in the testing of drug action, cytotoxic agents, and screening other biologically active compounds. Several methods can be used for such determinations, but indirect approaches using fluorescent or chromogenic indicators provide the most rapid and large-scale assays (Lindhagen et al., 2008). Among such procedures, the MTT assay is still among one of the most versatile and popular assays (Mosmann, 1983), and such a test was applied on the resultant AA. However, only limited attempts, have been made to evaluate the biosafety of AA resulted from keratin biodegradation. The increase in the viability of HepG2 cells without any morphological alteration supports the non-toxic action of the tested molecules (Wang et al., 2002). Although the current result is preliminary, it suggested that under *in vitro* conditions, the cell line of HepG2 was devoid of toxicity in mammalian cells at the tested concentration range and, therefore, may be considered safe from the nutritional point of view. However, a further *in vivo* study using animal models is encouraged to explore the possibility of AA’s allergenic and/or immune-modulatory reactions.

Summing up, the current study reported *B. paramycoides* ZW-5 as a new tool for the biodegradation of CFK into AA. The bioremediation process was mediated through RCCD and ANN; the latter is first reported here and proves its significance with higher accuracy and predictive ability than the RCCD. The SSF process of the CFK by *B. paramycoides* ZW-5 was carried out on a simple medium and produced 14 essential and non-essential AA. The toxicity test of AA on HepG2 cells showed no morphological alteration, encouraging further tests on animal models.

## Data Availability Statement

The original contributions presented in the study are included in the article/Supplementary Material, further inquiries can be directed to the corresponding author/s.

## Author Contributions

ZM and WS contributed to the conception and design of the study. ZM and SA organized the database. DD and WS performed the statistical analysis. ZM, SA, and DD wrote the first draft of the manuscript. ZM, DD, SA, and WS wrote sections of the manuscript. All authors contributed to manuscript revision, read, and approved the submitted version.

## Conflict of Interest

The authors declare that the research was conducted in the absence of any commercial or financial relationships that could be construed as a potential conflict of interest.

## Publisher’s Note

All claims expressed in this article are solely those of the authors and do not necessarily represent those of their affiliated organizations, or those of the publisher, the editors and the reviewers. Any product that may be evaluated in this article, or claim that may be made by its manufacturer, is not guaranteed or endorsed by the publisher.

## Footnotes

1
https://blast.ncbi.nlm.nih.gov/Blast.cgi

## Abbreviations

AA: amino acids
ANN: artificial neural network
CFK: chicken feather keratin
RCCD: rotatable central composite design
SSF: solid-state fermentation.
