# Supplementary material for: Innovative Artificial-Intelligence- Based Approach for the Biodegradation of Feather Keratin by Bacillus paramycoides, and Cytotoxicity of the Resulting Amino Acids
Source: Front Microbiol. 2021 Oct 22;12:731262. doi: 10.3389/fmicb.2021.731262 (PMC8569898; doi:10.3389/fmicb.2021.731262)
Supplement: Supplementary file 1 [file Table_1.DOCX]

Supplementary Material

Innovative artificial-intelligence-based approach for the biodegradation of feather keratin by *Bacillus paramycoides*, and cytotoxicity of the resulting amino acids

**Zeiad Moussa^1^, Doaa B. Darwish^2,3^, Salma S. T. Alrdahe^3^,** **WesamEldin I. A. Saber^1^***

^1^Microbial Activity Unit, Microbiology Department, Soils, Water and Environment Research Institute, Agricultural Research Center, Giza, Egypt

^2^Botany Department, Faculty of Science, Mansoura University, Mansoura, Egypt.

^3^Department of Biology, Faculty of Science, University of Tabuk, Tabuk, Saudi Arabia

*** Correspondence:**WesamEldin I. A. Saber
[wesameldin.saber@arc.sci.eg](mailto:wesameldin.saber@arc.sci.eg)

Keywords: keratinase, bioremediation, biodegradation, artificial neural network, mineral salts, artificial intelligence, response surface methodology

**Supplementary Table 1.** Analysis of variance, estimated effect, and regression coefficient (coded) of the total amino acids produced by *B. paramycoides* ZW-5 based on the experimental data of Plackett-Burman matrix.

| Term | | Estimated effect | Regression coefficient | Freedom degree | Contribution, % | F value | *P* value |
| --- | --- | --- | --- | --- | --- | --- | --- |
| Constant | | - | 655.2 | - | - | - | - |
| Single term | K_2_HPO_4_ | -98.5 | -49.2 | 1 | 1.2 | 3.1 | 0.139^ns^ |
|  | KH_2_PO_4_ | -289.8 | -144.9 | 1 | 10.6 | 26.7 | 0.004^*^ |
|  | NaCl | 120.8 | 60.4 | 1 | 1.9 | 4.6 | 0.084^ns^ |
|  | NH_4_Cl | 802.9 | 401.4 | 1 | 81.7 | 205.1 | < 0.001^*^ |
|  | MgSO_4_ | -53.4 | -26.7 | 1 | 0.4 | 0.9 | 0.385^ns^ |
|  | CaCl_2_ | 132.2 | 66.1 | 1 | 2.2 | 5.6 | 0.065^ns^ |
| Model | Linear |  |  | 6 | 98 | 41 | < 0.001^*^ |
|  | Error |  |  | 5 | 2 |  |  |
|  | Total |  |  | 11 | 100 |  |  |
| **The goodness-of-fit statistics of the model** | | | | | | | |
| Standard deviation | | | | | | 97.11 | |
| Coefficient of determination (R^2^) | | | | | | 98.01 | |
| Adjusted-R^2^ | | | | | | 95.62 | |
| Predicted-R^2^ | | | | | | 88.52 | |

* significant term, ^ns^ insignificant term.
